# Bone regeneration of demineralized dentin matrix with platelet-rich fibrin and recombinant human bone morphogenetic protein-2 on the bone defects in rabbit calvaria

**DOI:** 10.1186/s40902-021-00320-8

**Published:** 2021-09-09

**Authors:** Beom-Jin Kim, Seok-Kon Kim, Jae-Hoon Lee

**Affiliations:** 1 Seoul Boston Dental Clinic, 3rd Floor, Geumgang Plaza, 49, Cheongsa-ro, Uijeongbu-si, Gyeonggi-do Republic of Korea; 2 Department of Anesthesiology and Pain Medicine, College of Medicine, Dankook University, 201, Manghyang-ro, Dongnam-gu, Cheonan-si, Chungcheongnam-do Republic of Korea; 3 Department of Oral and Maxillofacial Surgery, College of Dentistry, Dankook University, 119, Dandae-ro, Dongnam-gu, Cheonan-si, Chungcheongnam-do 31116 Republic of Korea

**Keywords:** Demineralized dentin matrix (DDM), Platelet-rich fibrinogen (PRF), Recombinant human bone morphogenetic protein-2 (rhBMP-2)

## Abstract

**Background:**

This study was to evaluate the bone formation ability of demineralized dentin matrix (DDM) combined with platelet-rich fibrinogen (PRF) and DDM combined with recombinant human bone morphogenetic protein-2 (rhBMP-2) to improve the osteoinductive ability of DDM.

**Methods:**

After four bone defects with a diameter of 8mm were created in the calvarium of each rabbit, DDM was grafted into the first defect (experimental groups 1), a combination of DDM and PRF was grafted into the second defect (experimental groups 2), and DDM with absorbed rhBMP-2 was grafted into the third defect (experimental groups 3). The fourth defect was used as the control group. Twelve healthy male rabbits (New Zealand, white rabbits) weighing around 3.0–4.0 kg were used. Among 12 rabbits, 3 rabbits were sacrificed immediately after surgery and at 2, 4, and 8 weeks after surgery, respectively. Histopathologic analysis and histomorphometric analysis were conducted to evaluate bone formation in each group.

**Results:**

The PRF/DDM group did not show a significantly higher degree of new bone formation in calvarial bone defects than the DDM group at 2, 4, and 8 weeks postoperatively in histopathological findings and histomorphometric results. On the other side, the rhBMP-2/DDM group showed higher degrees of new bone formation and calcification, and the lamellae of bone matrix, which are observed in mature bone tissue, were more distinctly visible in the rhBMP-2/DDM group. Moreover, the rhBMP-2/DDM group showed a significantly higher amount of new bone formation, compared to the DDM group at 4 and 8 weeks postoperatively (*P*<0.05) in histomorphometric results.

**Conclusion:**

The DDM has great potential as a carrier for the maintenance and sustained release of rhBMP-2, which has been recently receiving wide attention as a type of signaling molecules to promote bone formation.

## Background

Continuous research efforts have been made to develop alternatives to existing bone grafting materials or to improve the bone formation ability of existing bone grafting materials.

Demineralized dentin matrix (DDM), which is a material produced by extracting bone components from extracted autogenous teeth, is a graft material developed based on the fact that teeth and bones are almost identical in chemical composition [1–4].

In addition, there are signaling molecules to promote the bone formation ability, such as platelet-derived growth factors (PDGF) [5, 6], bone morphogenetic proteins (BMP) [7], transforming growth factors (TGF-β) [8], epidermal growth factors (EGF), and fibroblast growth factors (FGF), and studies on these materials have been actively conducted.

Platelet-rich plasma (PRP) has been used in the treatment of thrombocytopenia, and as platelet concentrates have been locally applied to surgical sites, PRP is currently used as a term which refers to all types of autologous plasma including a platelet concentrate. Earlier, fibrinogen contained in PRP was obtained from human plasma of one or multiple donors, so there was a risk of HIV infection or hepatitis C transmission. In addition, thrombin, which is used in the production process of fibrin glue, is also derived from cattle, so the risk of transmission of diseases, including mad cow disease, has been reported [9, 10]. To address these risks, platelet-rich fibrin (PRF), in which autologous thrombin is concentrated in autologous fibrinogen without the addition of bovine thrombin or an anticoagulant agent, started to be used [11–13].

The bone morphogenetic protein (BMP) refers to a group of peptide growth factors belonging to the superfamily of transforming growth factors (TGFs) and acts on interstitial cells in mammals to promote cell differentiation into osteocytes and chondrocytes. In 1965 and 1971, Urist [14, 15] extracted the substance from the demineralized bone matrix and named it bone morphogenetic protein (BMP). To date, 15 types of BMPs from BMP-1 to BMP-15 have been identified, and among them, BMP-2, BMP-3, BMP-4, BMP-6, and BMP-7 have been found to induce bone formation [16, 17]. In particular, BMP-2 increases the activity of alkaline phosphatase and has been shown to effectively stimulate bone formation experimentally and clinically in the bone defect areas of the oral and maxillofacial region by enhancing the expression of bone marker genes [18, 19]_._

Therefore, this study aimed to evaluate the bone formation ability of human demineralized dendritic matrix combined with platelet-rich fibrin (PRF) and recombinant human bone morphogenetic protein-2 (rhBMP-2) in rabbit calvarial bone defects by histopathological and histomorphometric analyses.

## Materials and methods

### Subjects and materials

#### Experimental animals

Twelve healthy male rabbits (New Zealand, white rabbits) which weighed around 3.0–4.0 kg were included in the experiments. This study was approved by Kronex Co., Ltd. Animal Experimental Ethics Committee. The approval number of the animal experiment is CRONEX-IACUC:2013005.

#### Bone grafting materials

The extracted teeth were immersed in 70% ethyl alcohol and were delivered to a specialized treatment company, the Korea Tooth Bank Co. (Seoul, Korea), and separated into the crowns and roots after removing foreign materials such as soft tissue and plaque attached to each tooth, and then pulverization of the teeth was performed. After immersing 1–2-mm pulverized particles into distilled water and a hydrogen oxide solution, they were washed with an ultrasonic cleaner to remove the remaining foreign materials, and washed particles were dehydrated, followed by defatting using ethyl alcohol. After the procedure described above, the particles underwent lyophilization and were sterilized with ethyleneoxide gas, and they arrived at the lab in a wrapped container before they were used for bone grafting.

#### Signaling molecules to promote the bone formation ability

##### Preparation of platelet-rich fibrin (PRF)

When the rabbit was restrained so that it would not move, 8 ml of blood was collected directly from the ear with a syringe, and then 8 cc of blood was centrifuged immediately without putting it in a separate container for the addition of any substance or manipulation. Blood samples were centrifuged at 400G for 10 min (BMS, Korea).

##### Preparation of recombinant human bone morphogenetic protein-2 (rhBMP-2)

The recombinant human bone morphogenetic protein-2 (rhBMP-2) (Cowellmedi Co, Korea) with a concentration of 2mg/ml was added to 0.03g of DDM by the dip dry method and kept in a sterile container in the frozen state.

### Methods

#### Surgery of experimental animals

**Fig. 1:**
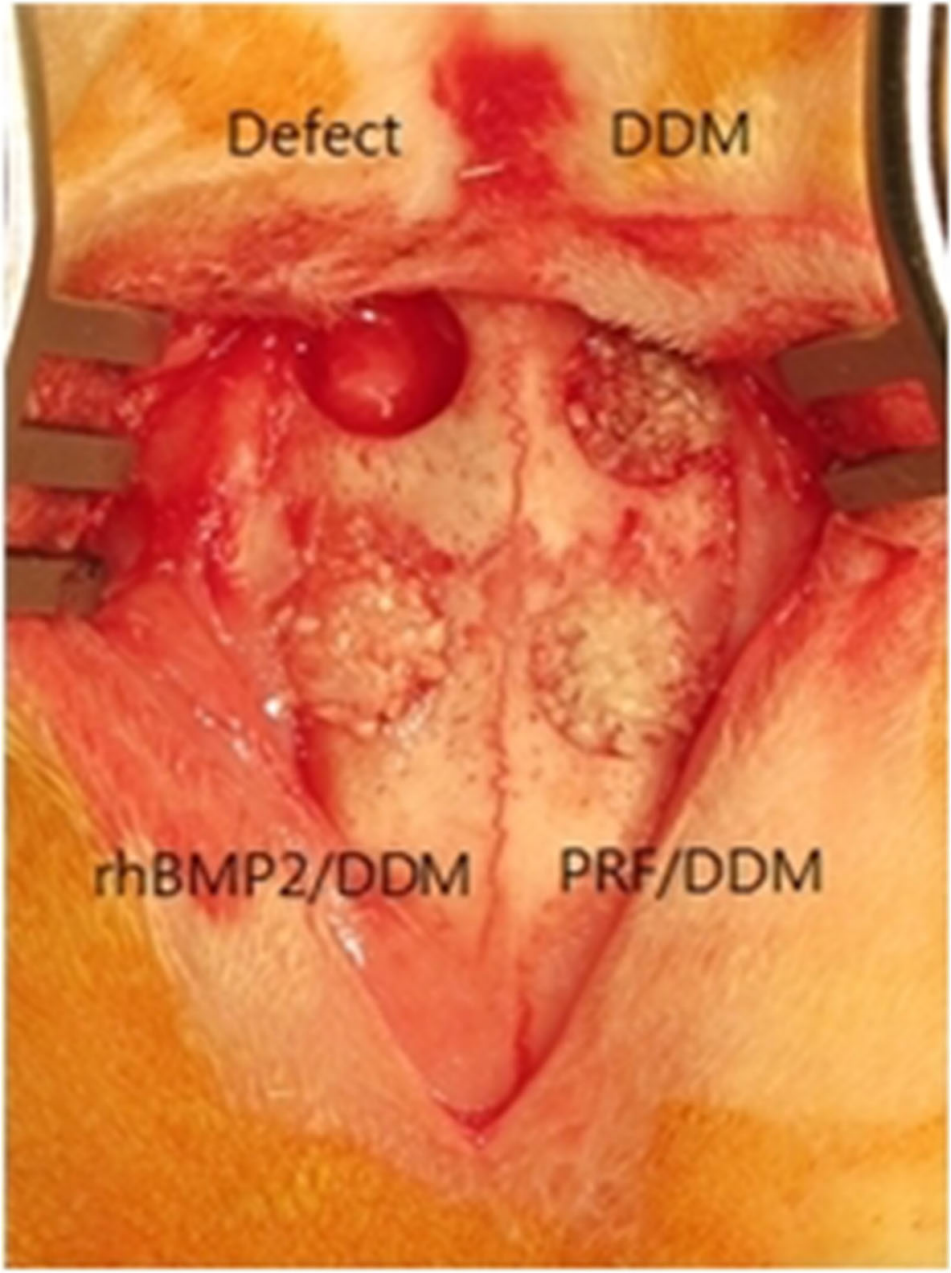
Experimental design. The control group received manual pressure hemostasis with gauze and did not receive any graft into the calvarial defect. In experimental group 1, bone defects were treated using DDM as a sole grafting material. Experimental group 2 were treated with a mixture of PRF and DDM. Experimental group 3 were treated using DDM with absorbed rhBMP-2

Each rabbit received an intramuscular injection of Zoletil 50 (0.5ml/kg) (Virvac, France) and Xilazine (0.25mg/kg) (Rompum, Bayer, Leverkusen, Germany) for general anesthesia. The calvarium was exposed by making a median incision in the skin of the scalp from the anterior part of the rabbit’s frontal bone to the posterior part. A circular defect of 8 mm in diameter and 3 mm in depth was created in the exposed calvarium using a trephine bur with 8 mm inner diameter and 9 mm outer diameter (3i, Palm Beach Gardens, FL, USA). In the control group, manual pressure hemostasis was performed with gauze without any treatment of the circular defect, and in experimental group 1, calvarial bone defects were treated using DDM as a sole grafting material. In experimental group 2, bone defects were treated with a mixture of PRF and DDM. In experimental group 3, bone defects were treated using DDM with absorbed rhBMP-2 (Fig. 1). Then, after covering the bone defect with the scalp skin, suturing was performed using 4-0 coated Vicryl® sutures (Polyglactin 910, braided absorbable suture, Ethicon, Johnson & Johnson Int., Edinburgh, UK), and sutures were removed 1 week after surgery.

#### Histopathological analysis

To make demineralized samples, three rabbits were sacrificed immediately after surgery and at 2, 4, and 8 weeks postoperatively, respectively, then the skull was detached, and tissue sections of surgical sites were obtained with a hard tissue cutting machine. After the collected tissue slices were sufficiently fixed with 70% ethyl alcohol and washed with running water, fixation was performed with 10% neutral formalin for the preservation of proteins in the tissue. Then, the tissues were dehydrated in 5% nitric acid solution for 5 days and they were embedded in paraffin. Finally, an incision was made to a thickness of 3 μm in a transverse direction of the calvarium, and after hematoxylin and eosin (H&E) staining and Masson’s trichrome (M-T) staining, histological examination was carried out using an Olympus BX-51 optical microscope (Olympus Co., Tokyo, Japan). The stained tissue slides were made with a digital camera attached to a scientific microscope, using the SPOT Advanced^TM^ software program (Olympus Co., Tokyo, Japan)

#### Histomorphometric analysis

At 2, 4, and 8 weeks after surgery, two tissue slides for each of the control and experimental groups 1, 2, and 3 were made. Microscopic images of the surgical sites were taken by randomly selecting four areas of the center portion of the defect at 100× magnification. Then, using 24 images of each group per week, the ratio of newly formed mineralized bone (new bone formation) to the entire defect area in the slide images was calculated using the Image Pro plus® image analysis program. The area of newly formed bone was defined as the area up to the boundary of newly formed bone including mineralized bone, fibrous connective tissue, and new blood vessels. Statistical analysis was carried out using SSPS ver. 17.0 (SPSS, Chicago, IL, USA). A Mann-Whitney *U* test was conducted, and the level of significance was defined as *P* < 0.05.

## Results

### Histopathological findings

#### The control group

##### Findings at 2 weeks

**Fig. 2:**
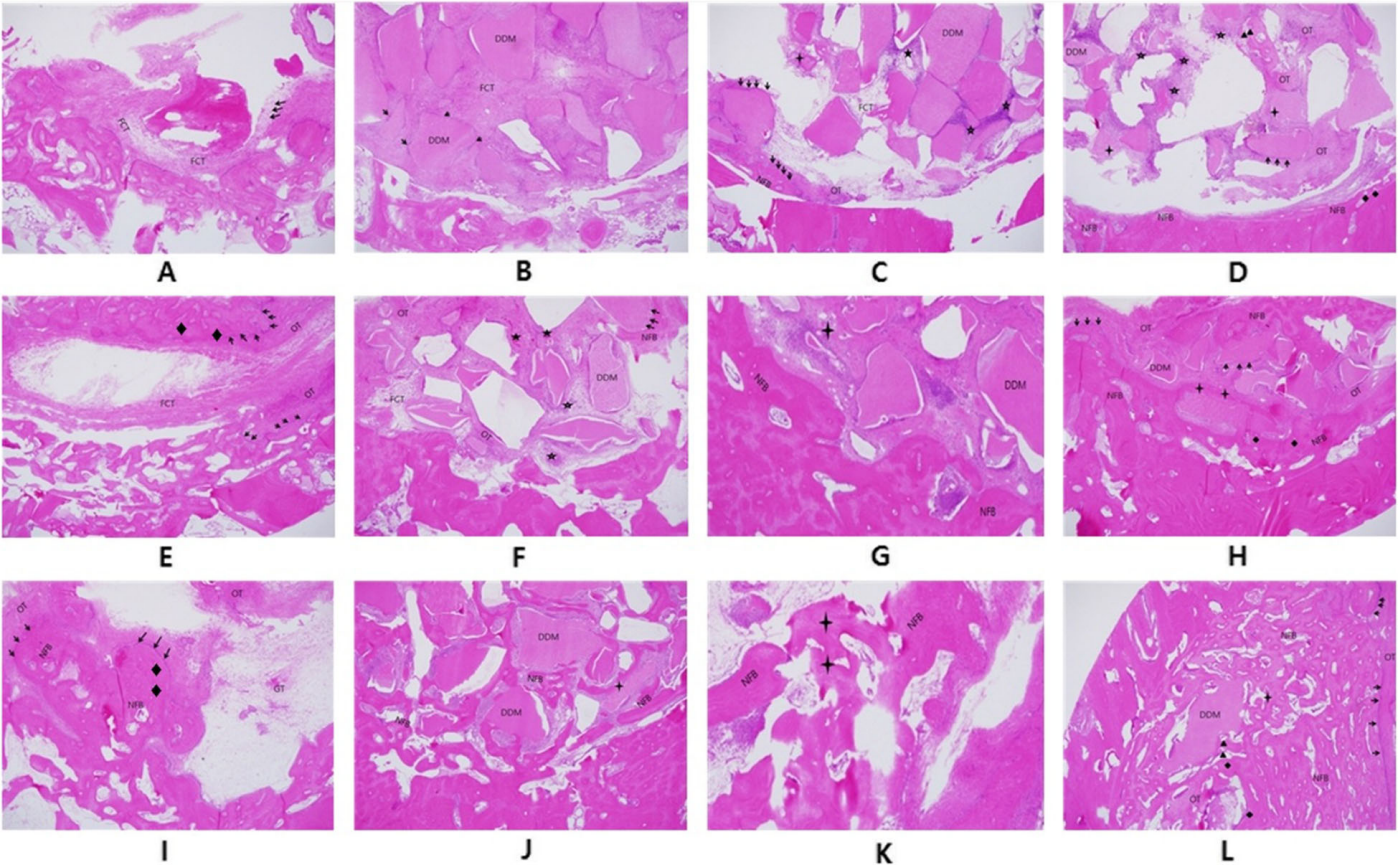
Hematoxylin and eosin stain (original magnification ×40). **A** Histologic finding of the control group after 2 weeks (FCT fibrous connective tissue). **B** Histologic finding of the DDM group after 2 weeks (→ osteoblast, FCT fibrous connective tissue). **C** Histologic finding of the PRF/DDM group after 2 weeks (→ osteoblast, OT osteoid tissue, ★ blood vessel, FCT fibrous connective tissue, NFB newly formed bone). **D** Histologic finding of the rhBMP2/DDM group after 2 weeks (→ osteoblast, OT osteoid tissue, ☆ cluster of fibroblast, ◆ osteocyte, : resorbed DDM, ▲ osteoclast, NFB newly formed bone). **E** Histologic finding of the control group after 4 weeks (→ osteoblast, ◆ osteocyte, OT osteoid tissue, FCT fibrous connective tissue). **F** Histologic finding of the DDM group after 4 weeks (→ osteoblast, OT osteoid tissue, ★ blood vessel, ☆ cluster of fibroblast, FCT fibrous connective tissue, NFB newly formed bone). **G** Histologic finding of the PRF/DDM group after 4 weeks (: resorbed DDM, OT osteoid tissue, NFB newly formed bone). **H** Histologic finding of the rhBMP2/DDM group after 4 weeks (→ osteoblast, OT osteoid tissue, ◆ osteocyte, : resorbed DDM, NFB newly formed bone). **I** Histologic finding of the control group after 8 weeks (→ osteoblast, ◆ osteocyte, GT granulation tissue, NFB newly formed bone). **J** Histologic finding of the DDM group after 8 weeks (NFB newly formed bone). **K** Histologic finding of PRF/DDM group after 8 weeks (: resorbed DDM, NFB newly formed bone). **L** Histologic finding of the rhBMP2/DDM group after 8 weeks (: resorbed DDM, → osteoblast, OT osteoid tissue, ◆ osteocyte, : resorbed DDM, ▲ osteoclast, NFB newly formed bone)

**Fig. 3:**
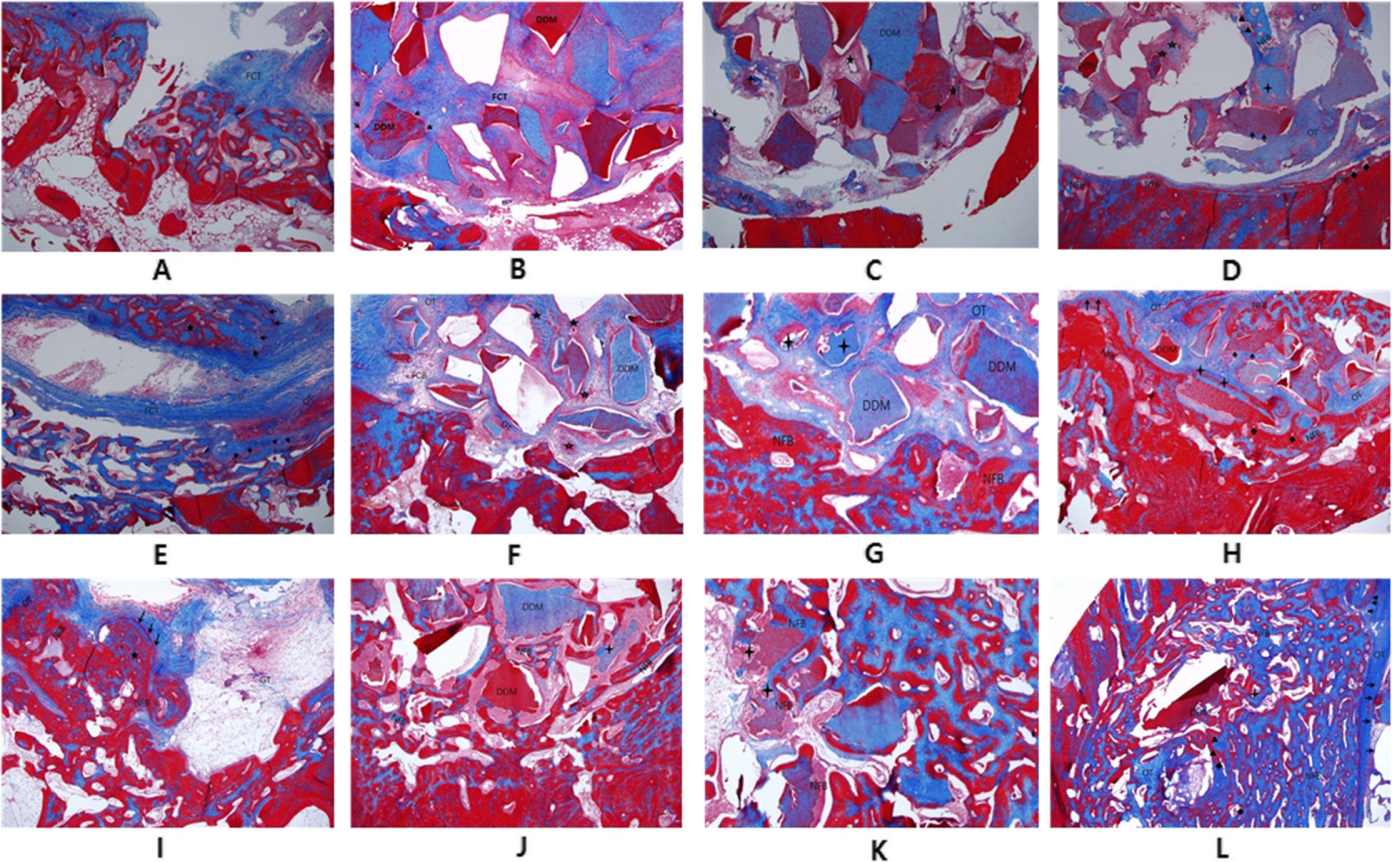
MT stain (original magnification ×40). **A** Histologic finding of the control group after 2 weeks (FCT fibrous connective tissue). **B** Histologic finding of the DDM group after 2 weeks (→ osteoblast, FCT fibrous connective tissue). **C** Histologic finding of the PRF/DDM group after 2 weeks (→ osteoblast, OT osteoid tissue, ★ blood vessel, : resorbed DDM, FCT fibrous connective tissue, NFB newly formed bone). **D** Histologic finding of the rhBMP2/DDM group after 2 weeks (→ osteoblast, OT osteoid tissue, ☆ cluster of fibroblast, ◆ osteocyte, : resorbed DDM, ▲ osteoclast, NFB newly formed bone). **E** Histologic finding of the control group after 4 weeks (→ osteoblast, ★ osteocyte, OT osteoid tissue, FCT fibrous connective tissue). **F** Histologic finding of the DDM group after 4 weeks (→ osteoblast, OT osteoid tissue, ★ blood vessel, ☆ cluster of fibroblast, FCT fibrous connective tissue, NFB newly formed bone). **G** Histologic finding of the PRF/DDM group after 4 weeks (: resorbed DDM, OT osteoid tissue, NFB newly formed bone). **H** Histologic finding of the rhBMP2/DDM group after 4 weeks (→ osteoblast, OT osteoid tissue, ◆ osteocyte, : resorbed DDM, NFB newly formed bone). **I** Histologic finding of the control group after 8 weeks (→ osteoblast, ★ osteocyte, GT granulation tissue, NFB newly formed bone). **J** Histologic finding of the DDM group after 8 weeks (: resorbed DDM, NFB newly formed bone). **K** Histologic finding of the PRF/DDM group after 8 weeks (: resorbed DDM, NFB newly formed bone). **L** Histologic finding of the rhBMP2/DDM group after 8 weeks (→ osteoblast, OT osteoid tissue, ◆ osteocyte, : resorbed DDM, ▲ osteoclast, NFB newly formed bone)

The defect was filled with irregular, thin, fibrous connective tissue, and bleeding was observed. New bone was not detected (Figs. 2A and 3A).

##### Findings at 4 weeks

Partially aligned connective tissues were observed in the defect, a minimal degree of new bone formation and osteoid tissue began to appear in the defect margin, and osteocytes were observed along the defect margin (Figs. 2E and 3E).

##### Findings at 8 weeks

Relatively well-aligned connective tissue with uniform density was observed in the defect, and granulation tissue was detected in the center of the defect. New bone formation was identified only in the defect margin, but features of immature bones such as a large medullary cavity were found (Figs. 2I and 3I).

#### Experimental group 1 (the DDM group)

##### Findings at 2 weeks

The defect area was mostly filled with DDM, and fibrous connective tissues with osteoblasts were observed among the DDM particles (Figs. 2B and 3B).

##### Findings at 4 weeks

The degree of new bone formation in the defect margin was higher compared to the control group. Osteoid tissue was also detected across the defect area, and bone formation was actively progressing (Figs. 2F and 3F).

##### Findings at 8 weeks

New bone was formed as a large portion of the DDM which filled the defect area was absorbed. As bone maturation and bone remodeling progressed in the defect margin, new bone and existing bone were found to coexist. As the calcification of newly formed bone progressed further, bony trabecula became more dense and a mature pattern of bony trabecula similar to that of the normal bone was observed. Compared to the control group, osteoinduction was observed (Figs. 2J and 3J).

#### Experimental group 2 (the PRF/DDM group)

##### Findings at 2 weeks

The area around DDM particles was found to be surrounded by a large amount of osteoblasts, and particles of absorbed DDM were also observed along with osteoclasts. New bone formation was actively progressing in the defect margin and neovascularization was observed among DDM particles (Figs. 2C and 3C).

##### Findings at 4 weeks

Compared with experimental group 1, a large amount of new bone formation was observed in the defect margin, and absorbed DDM particles were found together with osteoclasts. Osteoid tissue was observed among DDM particles, indicating that bone formation was actively progressing (Figs. 2G and 3G).

##### Findings at 8 weeks

Compared with experimental group 1, new bone was formed farther from the defect margin, new bone extended into the area where collagen bundles and DDM were completely absorbed, and gradual calcification was also observed. In particular, as osteoid tissue and cancellous bone decreased, the lamellae of bone matrix, which are observed in mature bone tissue, were observed. Compared with the control group, osteoinduction was observed (Figs. 2K and 3K).

#### Experimental group 3 (the rhBMP-2/DDM group)

##### Findings at 2 weeks

New bone formation was observed not only in the defect margin but also in the central part of the defect. Absorbed DDM particles along with more osteoclasts were observed compared to experimental group 2 (Figs. 2D and 3D).

##### Findings at 4 weeks

Compared to experimental group 2, the degrees of new bone formation and bone remodeling were higher, showing that they progressed faster, and it was found that bone tissue formation and calcification were gradually progressing in the way that bone tissue was surrounded by a newly formed bone matrix in the periphery of DDM, the absorbed bone graft material (Figs. 2H and 3H).

##### Findings at 8 weeks

A large amount of new bone formed not only in the defect margin but also in the entire defect area including the central part of the defect. The degree of new bone formation and calcification were higher than experimental group 2, showing that they were progressing faster. Also, the lamellae of bone matrix, which are observed in mature bone tissue, were more distinctly visible. Compared to the control group, osteoinduction was observed (Figs. 2L and 3L).

### Histomorphometric results

The amount of new bone formation in the calvarial defect was 3.2 observed in ma, and 8.1nt of new bon, and 8 weeks, respectively, in the control group, and 20.3as 3.2 observed in mature bone tissue, we, and 8 weeks, respectively, in the DDM group. Also, it was 22.7±10.2%, 31.2±11.7%, and 46.5±15.1% at 2, 4, and 8 weeks, respectively, in the PRF/DDM group, and 26.9 22.7±10.2%, 31.2±11.7%,58.7 8 weeks, resp, and 8 weeks, respectively, in the rhBMP-2/DDM group. The measurements of new bone formation showed that there was not a statistically significant difference between the PRF/DDM group and the DDM group at 2, 4, and 8 weeks. (Table 1). On the other side, there was a statistically significant difference between the rhBMP-2/DDM group and the DDM group at 4 and 8 weeks (*P* < 0.05) (Table 1).

**Table 1:** New bone formation

Group	2 weeks	4 weeks	8 weeks
**Control group (defect)**	**3.2fect**	**5.8fect**	**8.1fect**
**Experimental group 1 (DDM)**	**20.3rime**	**30.6rimen**	**45.1rimen**
**Experimental group 2 (PRF/DDM)**	**22.7rimen**	**31.2rimen**	**46.5rimen**
**Experimental group 3 (rhBMP-2/DDM)**	**26.9rimen**	**42.7±13.8**★	**58.7±19.8**★

^★^Statistically significant difference compared to the DDM group (*P* < 0.05) (each group averagefstandard deviation, %)

## Discussion

The rabbit calvarial defect model is known to be useful for analyzing and evaluating the bone formation ability of bone grafting materials because it has better accessibility and reproducibility than other animal models and is easy to handle. The calvarial bone consists of inner and outer tables (cortical bone) and trabecular bone between them, so it has the advantage that it is biomechanically similar to human jaw bones. Experiments were conducted under the same conditions and rabbits were kept under the same conditions, including solid feed and separate indoor rooms (average room temperature of 22°C, light-dark cycles of 12:12 h). Twelve male rabbits were used as subjects and female rabbits were excluded from the experiment because hormonal changes or pregnancy of females may affect the experimental results. Circular bone defects with a diameter of 8 mm, which is the critical size defect (CSD) in rabbits, were created in the calvaria. The CSD refers to the smallest size defect in an animal which shows less than 10% bone regeneration when left untreated [20]. In addition, when resected bone tissue is removed after creating a circular bone defect with a diameter of 8 mm and a depth of 3 mm using a trephine bur with 8 mm inner diameter and 9 mm outer diameter (3i, Palm Beach Gardens, FL, USA), the cranial dura mater of the rabbit is sometimes exposed. In such cases, care was taken to preserve the cranial dura mater because it plays an important role in the bone formation of bone graft materials.

Tooth ash powder, a previously developed bone grafting material reported in prior studies, is produced by pulverizing extracted tooth into powder after high-temperature treatment to inhibit immune responses against bone grafts, so it has been reported to exhibit only osteoconductive capacity because it consists only of minerals. In contrast, DDM used in this study contained many bone growth factors, including type I collagen and BMP preserved in the dentin of teeth, and thus was shown to exhibit osteoinductive capacity as well as osteoconductive capacity. The histological analysis of this study also showed that osteoinduction occurred in all the experimental groups, different from the control group. In all the experimental groups, osteoblasts gradually formed from bone tissue toward DDM, the bone grafting material, and at the same time, new bone tissue was formed directly from the peripheral area of DDM.

In the present study, histomorphometric analysis of the amount of new bone formation did not show statistically significant differences between the PRF/DDM group and the DDM group. New bone formation was observed farther away from the defect margin in the PRF/DDM group, compared to the DDM group. Moreover, new bone extended into the area where collagen bundles and DDM were absorbed and gradual mineralization was observed in the PRF/DDM group.

In 1965 and 1971, Urist [14, 15] reported that heterotrophic ossification occurred around the bone graft material when the demineralized bone flap was transplanted in the subcutaneous tissue of rats, and the specific type of proteins in bone tissue which contribute to bone formation was named bone morphogenetic protein (BMP). In 2019 and 2020, BMP-2 increases the activity of alkaline phosphatase and has been shown to effectively stimulate bone formation experimentally and clinically in the bone defect areas of the oral and maxillofacial region by enhancing the expression of bone marker genes [18, 19]_._

In this study, histological analysis showed that in contrast to the DDM group, the PRF/DDM group and the rhBMP-2/DDM group showed new bone was directly formed from the peripheral area of DDM. The PRF/DDM group did not show a significantly higher degree of new bone formation in bone defects than the DDM group at 2, 4, and 8 weeks postoperatively.

On the other side, a larger amount of new bone formed in the entire defect area including the central part of the defect as well as the defect margin in the rhBMP-2/DDM group, compared to the DDM group. Also, the rhBMP-2/DDM group showed higher degrees of new bone formation and calcification, and the lamellae of bone matrix, which are observed in mature bone tissue, were more distinctly visible in the rhBMP-2/DDM group. Moreover, the histomorphometric analysis revealed that the amount of new bone formation in the calvarial defect area was significantly higher in the rhBMP-2/DDM group than in the DDM group at 4 and 8 weeks after surgery (*P* < 0.05).

## Conclusion

Demineralized dentin matrix (DDM) is a material with great potential as an excellent carrier for the maintenance and controlled-release of recombinant human bone morphogenetic protein-2 (rhBMP-2), which has been recently been receiving wide attention as a type of signaling molecules to promote bone formation ability.

## Data Availability

Data sharing is not applicable to this article since no dataset was generated or analyzed during the current study.

## Abbreviations

DDM: Demineralized dentin matrix
rhBMP-2: Recombinant human bone morphogenetic protein-2
PRF: Platelet-rich fibrinogen
PDGF: Platelet-derived growth factors
BMP: Bone morphogenetic proteins
EGF: Epidermal growth factors
FGF: Fibroblast growth factors
PRP: Platelet-rich plasma
FDBA: Freeze-dried bone allograft
DFDBA: Demineralized freeze-dried bone allograft
CSD: Critical size defect
H&E staining: Hematoxylin and eosin staining
M-T staining: Masson’s trichrome staining
TGF: Transforming growth factors
